# A comparison of the diagnostic value of MRI and ^18^F-FDG-PET/CT in suspected spondylodiscitis

**DOI:** 10.1007/s15010-016-0914-y

**Published:** 2016-06-17

**Authors:** Carolijn Smids, Ilse J. E. Kouijzer, Fidel J. Vos, Tom Sprong, Allard J. F. Hosman, Jacky W. J. de Rooy, Erik H. J. G. Aarntzen, Lioe-Fee de Geus-Oei, Wim J. G. Oyen, Chantal P. Bleeker-Rovers

**Affiliations:** 1 Department of Internal Medicine, Radboud University Medical Center, P.O. Box 9101, 6500 HB Nijmegen, The Netherlands; 2 Sint Maartenskliniek, P.O. Box 9011, 6500 GM Nijmegen, The Netherlands; 3 Department of Internal Medicine, Canisius-Wilhelmina Hospital, P.O. Box 9015, 6500 GS Nijmegen, The Netherlands; 4 Department of Orthopaedics, Radboud University Medical Center, P.O. Box 9101, 6500 HB Nijmegen, The Netherlands; 5 Department of Radiology and Nuclear Medicine, Radboud University Medical Center, P.O. Box 9101, 6500 HB Nijmegen, The Netherlands; 6 Department of Nuclear Medicine, Leiden University Medical Center, P.O. Box 9600, 2300 RC Leiden, The Netherlands; 7 MIRA Institute for Biomedical Technology and Technical Medicine, Biomedical Photonic Imaging Group, University of Twente, P.O Box 217, 7500 AE Enschede, The Netherlands

**Keywords:** Spondylodiscitis, MRI, ^18^F-FDG-PET/CT, Abscess

## Abstract

**Purpose:**

The purpose of this study was to evaluate the diagnostic value of ^18^F-fluorodeoxyglucose (FDG) positron emission tomography and computed tomography (PET/CT scan) and magnetic resonance imaging (MRI) in diagnosing spondylodiscitis and its complications, such as epidural and paraspinal abscesses.

**Methods:**

From January 2006 to August 2013 patients with a clinical suspicion of spondylodiscitis, with an infection, or with fever of unknown origin were retrospectively included if ^18^F-FDG-PET/CT and MRI of the spine were performed within a 2-week time span. Imaging results were compared to the final clinical diagnosis and follow-up data were collected.

**Results:**

Sixty-eight patients were included of whom 49 patients were diagnosed with spondylodiscitis. MRI showed an overall sensitivity of 67 % and specificity of 84 %. Diagnostic accuracy was 58 %, when MRI was performed within 2 weeks after the start of symptoms and improved to 82 %, when performed more than 2 weeks after onset of symptoms. ^18^F-FDG-PET/CT showed a sensitivity of 96 % and a specificity of 95 %, with no relation to the interval between the scan and the start of symptoms.

**Conclusions:**

As compared to MRI, ^18^F-FDG-PET/CT has superior diagnostic value for detecting early spondylodiscitis. After 2 weeks both techniques perform similarly.

## Introduction

The prevalence of spondylodiscitis, a severe infection of the spine, is increasing in our ageing society [1]. The entities spondylodiscitis, discitis, and vertebral osteomyelitis refer to the exact anatomic localization of the infection, but since diagnosis and management are usually identical, spondylodiscitis will be used in this article when referring to these types of infections. Common complications, which may result in paraplegia and other neurological damage, are epidural and spinal abscesses, and paravertebral or psoas abscesses. One-third of the patients suffer from residual spinal dysfunction or persistent pain after recovery [2, 3]. Thus, early and accurate detection is crucial for successful management and improved neurological outcome.

The diagnosis of spondylodiscitis is based on clinical, laboratory, and radiological features. Symptoms and signs of spondylodiscitis are often non-specific, so diagnosing spondylodiscitis is difficult in many patients. Spondylodiscitis is commonly diagnosed in patients with complicated bacteraemia [4]. Contrast-enhanced magnetic resonance imaging (CE-MRI) is the modality of choice in current clinical practice. Small studies investigating the value of magnetic resonance imaging (MRI) in diagnosing spondylodiscitis showed a sensitivity of 82–96 %, specificity of 85–93 %, and accuracy of 81–94 % [5–7].

Combined ^18^F-fluorodeoxyglucose (FDG) positron emission tomography and computed tomography (PET/CT) is increasingly used in the diagnostic workup of infectious diseases. Since ^18^F-FDG-PET/CT has been shown to be cost-effective in patients with Gram-positive bacteraemia [8], many of the patients suspected of spondylodiscitis after bacteraemia undergo ^18^F-FDG-PET/CT. No large studies comparing the diagnostic value of ^18^F-FDG-PET/CT and MRI in patients with a suspicion of spondylodiscitis have been performed. Small studies speculated that ^18^F-FDG-PET might have a higher sensitivity (up to 100 %) than MRI in diagnosing spondylodiscitis, especially in the early stages of disease [6, 9, 10]. This might be explained by the different features of both imaging modalities; MRI merely relies on anatomical changes, whereas ^18^F-FDG-PET visualizes glucose metabolism, which is already increased in the very early stages of inflammation. PET/MRI systems have been technically developed ahead of considering and identifying clinical applications. So, it is of importance to find indications where PET/MRI in a one-stop shop could benefit and where the unique features of combined PET/MRI have potential competitive advantages over PET/CT. Since there are only a limited number of studies available on this topic, the purpose of this study was to determine the diagnostic value of MRI and ^18^F-FDG-PET/CT in diagnosing spondylodiscitis and in diagnosing its complications, such as epidural, spinal, paravertebral, and psoas abscesses in a setting of clinical practice.

## Materials and methods

### Study design

Patients with a clinical suspicion of spondylodiscitis, systemic infection, or with fever of unknown origin in whom ^18^F-FDG-PET/CT was performed between January 2006 and August 2013 were identified using the electronic database of the Department of Radiology and Nuclear Medicine. Patients were included if MRI of the spine was performed within 2 weeks before or after ^18^F-FDG-PET/CT. Exclusion criteria were MRI or ^18^F-FDG-PET/CT done within 6 weeks after spinal surgery or known spinal fracture, or no established final clinical diagnosis. Patients from three hospitals were included: Radboud university medical center (Radboudumc), a tertiary referral center, Canisius-Wilhelmina Hospital, a community hospital, and Sint Maartenskliniek, a hospital specializing in musculoskeletal related disorders, all located in Nijmegen, The Netherlands.

The final clinical diagnosis served as standard of reference. Spondylodiscitis was considered to be the final clinical diagnosis in patients in whom a microorganism was isolated through biopsy, surgery, or from blood cultures in combination with compatible clinical and laboratory findings and/or imaging follow-up showing response to antibiotic therapy. According to the Dutch law, this study was exempt from approval by an ethics committee, because of the retrospective character of this study and the anonymous storage of data.

### Diagnostic workup

An integrated PET/CT scanner (Biograph or mCT; Siemens for Radboudumc and Sint Maartenskliniek, Philips TOF for Canisius-Wilhelmina Hospital) was used for obtaining data. Before ^18^F-FDG injection patients fasted and any glucose or insulin-containing infusions were discontinued for at least 6 h. At the time of ^18^F-FDG injection glucose was below 12 mmol/l in all patients, including diabetic patients. 1 h after intravenous injection of mean 200 MBq of ^18^F-FDG (Covidien, Petten, The Netherlands or IBA, Amsterdam, The Netherlands), a low-dose CT scan of the area between proximal femora and base of the skull was acquired for anatomic correlation and attenuation correction of the PET data. Emission images of the same area were acquired. Spinal infection on ^18^F-FDG-PET/CT was defined as increased ^18^F-FDG of the spine compared to uptake in bone marrow, or when ^18^F-FDG was increased in soft tissues around the spine. MRI with (55/68) or without (13/68) intravenous injection of gadolinium was performed on a 1.5 T Avanto MRI system; Siemens Erlangen, Germany in case of Radboudumc and Philips Intera 1.5 T in case of Sint Maartenskliniek, and Philips Achieva 1.5 T in case of Canisius-Wilhelmina Hospital. Spinal infection on MRI was defined as an affected intravertebral disc, or in case of narrowing of the intravertebral disc, or in case of present increased contrast in the spine. Because the purpose of this study was to investigate the value of ^18^F-FDG-PET/CT and MRI in spondylodiscitis in the daily clinical setting, the original clinical reports of ^18^F-FDG-PET/CT and MRI were used for this study. For this reason, no rereading by independent radiologists and nuclear physicians was performed.

### Patient follow-up

Follow-up data of patients at 3 and 6 months after presentation, as well as from the last available follow-up, were collected from medical charts. Results of diagnostic tests (blood cultures, other cultures, biopsies, C-reactive protein (CRP)) and treatment (surgery/antibiotic therapy) were obtained. Outcome was classified as recovery, still on treatment of the first episode of spondylodiscitis, relapse, neurological impairment, persistent back pain, or death (overall and infection-related). Relapse was defined as a second episode of spondylodiscitis with the same causative organism after completion of adequate antibiotic treatment of at least 6 weeks duration. Neurological impairment was defined as irreversible spinal cord injury, when available, classified according to the American Spinal Injury Association Impairment Scale, with motor weakness or sensory loss existing at last follow-up. Mortality at day 30 and overall was calculated. Mortality was considered to be infection-related when a patient died during the episode of spondylodiscitis with persistent signs or symptoms of systemic infection or after relapse.

### Statistical analysis

All data were collected in a structured database using SPSS statistics (version 20.0; IMB Corp.). Sensitivity, specificity, positive predictive value (PPV), negative predictive value (NPV), and accuracy were calculated with 95 % confidence intervals (CIs). Differences in outcomes were tested using Fisher’s exact test for categorical variables. Differences in continuous variables were tested with Mann–Whitney *U* test. Statistical significance was defined as a *p* value less than 0.05.

## Results

### Clinical features and diagnosis

Between January 2006 and August 2013, 75 eligible patients were identified. Three patients were excluded because MRI or ^18^F-FDG-PET/CT was done within 6 weeks after spinal surgery. Four patients were excluded because no final clinical diagnosis was established. Sixty-three patients were recruited from the Radboudumc and five from the other two hospitals. Baseline characteristics of the 68 study patients are provided in Table 1. Forty-nine patients (72 %) were diagnosed with spondylodiscitis. Fever (≥38 °C), back pain, elevated CRP, and positive blood cultures occurred significantly more often in the patients diagnosed with spondylodiscitis (Tables 1, 2).

**Table 1:** Baseline characteristics

	Final clinical diagnosis of spondylodiscitis	Final clinical diagnosis other than spondylodiscitis	*p* value
Total number of patients	49 (72 %)	19 (28 %)	–
Male	24 (49 %)	11 (58 %)	0.594
Median age (years)	64 (10–88)	65 (20–78)	0.795
Hospital admission	49 (100 %)	15 (79 %)	
Median duration of hospital admission in days (range)	46 (10–126)^a^	19 (7–67)	0.004
Fever (≥38 °C)	46 (94 %)	10 (53 %)	<0.001
Median duration of fever in days (range)	11.5 (1–136)	18 (5–60)	0.048
Back pain	47 (96 %)	12 (63 %)	0.001
CRP (mg/l)			
Median CRP at presentation (range)	188 (9–518)	64 (<5–396)	0.007
Median maximal CRP (range)	242 (9–518)	141 (<5–396)	0.004
Malignancy	9 (18 %)	5 (26 %)	0.512
Immunocompromised	9 (18 %)	6 (31 %)	0.329
Osteosynthesis material spine	1 (2 %)	1 (5 %)	0.484
Spinal surgery >6 weeks before imaging	5 (10 %)	2 (11 %)	1.000
Time between surgery and imaging in months	2, 41, 67, 468, 510	6, 297	–
Spine level^b^			
Cervical	14	–	–
Thoracic	18	–	–
Lumbar	31	–	–
Sacral	8	–	–

^a^One patient died during admission, 40 days after presentation

^b^Of all 49 patients, 21 had more than one affected spinal level

**Table 2:** Microbiological findings

	Final clinical diagnosis of spondylodiscitis	Final clinical diagnosis other than spondylodiscitis	*p* value
Blood cultures			
Positive	45 (92 %)	5 (26 %)^a^	<0.001
Median number of blood cultures taken (range)	11 (2–46)	6 (0–29)	0.001
Median duration of positive blood cultures in days (range)	4 (1–71)	3 (1–10)	0.832
Tissue cultures (biopsy)	12	–	–
Positive	5 (42 %)	–	–
Causative organism	46 (94 %)	–	–
*Staphylococcus aureus*	25 (51 %)	–	–
Coagulase-negative *staphylococcus*	2 (4 %)	–	–
*Streptococcus* species	11 (22 %)	–	–
*Escherichia coli*	2 (4 %)	–	–
*Candida* *albicans*	1 (2 %)	–	–
Polymicrobial infection	2 (4 %)^b^	–	–
Miscellaneous	3 (6 %)^c^	–	–
No causative organism	3 (6 %)	–	–

^a^ *S. aureus* (*n* = 3), *E. coli* (*n* = 1) and polymicrobial (*n* = 1, *S. aureus* and *Streptococcus milleri)*

^b^ *E. coli* and coagulase-negative *Staphylococcus*, *S. aureus* and *beta*-*hemolytic* group C *Streptococcus*

^c^ *Peptostreptococcus micros, Enterobacter cloacae* and *Enterococcus faecium*

Of all 49 patients with a clinical diagnosis of spondylodiscitis, a causative organism was identified in 46 patients. The most prevalent pathogens were *Staphylococcus aureus* (*n* = 25) and *Streptococcus* species (*n* = 11) (Table 2). The different ways the final clinical diagnoses of spondylodiscitis were established are shown in Table 3. Patients eventually not diagnosed with spondylodiscitis had degenerative spinal changes (*n* = 7), spinal disc herniation (*n* = 2), cauda equina syndrome (*n* = 1), metastatic neoplasm (*n* = 1), *S. aureus* meningitis (*n* = 1), spinal hemangioma (*n* = 1), osteomyelitis of the pelvis (*n* = 1), or back pain of unknown origin (*n* = 5).

**Table 3:** Establishing the final clinical diagnosis of spondylodiscitis

Final clinical diagnosis of spondylodiscitis	49
Positive cultures of spinal biopsy	5
CT-guided	1
Open	4
Vertebral biopsies showing osteomyelitis	2
Blood cultures and cultures from abscesses near the spinal level of infection positive for the same micro-organism^a^	4
Blood cultures and cultures from the cerebrospinal fluid positive for the same micro-organism^a^	2
Positive blood cultures^a^	33
Clinical symptoms in combination with imaging results and resolution of symptoms after treatment	3

^a^In combination with imaging results showing spondylodiscitis

### MRI

The overall sensitivity of diagnosing spondylodiscitis with MRI was 67 % with a specificity of 84 %, a PPV of 92 %, an NPV of 50 %, and a diagnostic accuracy of 72 %. Eleven of 16 false-negative MRIs were performed within 2 weeks of onset of symptoms (fever or back pain). In eight of these 16 patients, MRI was repeated, median 41 days after onset of symptoms (range 15–60) and median 22.5 days (range 9–46) after the first MRI. In one patient, this second MRI was of insufficient quality due to movement artifacts caused by claustrophobia. In the remaining 7 patients, the second MRI confirmed spondylodiscitis. The accuracy of MRI performed within 2 weeks after emerging of symptoms was 58 % compared to an accuracy of 82 % when MRI was performed more than 2 weeks after onset of symptoms (Table 4). In case of a prior probability of 50 % for spondylodiscitis, PPV was 82 % and NPV was 72.5 % for MRI. In case of a 30 % prior probability, the PPV is 62 % and the NPV is 83 % for MRI.

**Table 4:** Diagnostic value of MRI and ^18^F-FDG-PET/CT related to the timing of the imaging procedure

	≤ 14days of start of symptoms	>14 days of start of symptoms	Total
MRI	29	39	68
Sensitivity	50 (28.3–71.8)	82 (61.9–93.6)	67 (52.5–80.0)
Specificity	86 (42.2-97.6)	83 (51.6–97.4)	84 (60.4–96.4)
PPV	92 (61.5-98.6)	92 (73.0–98.7)	92 (77.5–98.2)
NPV	35 (14.3-61.7)	67 (38.4–88.1)	50 (31.9–68.1)
Accuracy	58 (40.0-76.0)	82 (69.9–94.1)	72 (61.3–82.7)
^18^F-FDG-PET/CT	32	36	68
Sensitivity	96 (79.6–99.3)	96 (78.8–99.3)	96 (86.0–99.4)
Specificity	100 (58.9–100)	92 (61.5–98.6)	95 (73.9–99.1)
PPV	100 (85.6–100)	96 (78.8–99.3)	98 (88.9–99.7)
NPV	88 (47.4–97.9)	92 (61.5–98.6)	90 (68.3–98.5)
Accuracy	97 (91.1–100)	94 (86.2–100)	96 (91.3–100)

In 13 patients (19 %), MRI was performed without intravenous contrast for various reasons including presumed allergy. Three (23 %) of the MRIs without contrast were false negative compared to 13 (24 %) of the contrast-enhanced MRIs.

### ^18^F-FDG-PET/CT

The overall sensitivity for diagnosing spondylodiscitis with ^18^F-FDG-PET/CT was 96 % with a specificity of 95 %, a PPV of 98 %, a NPV of 90 %, and an overall diagnostic accuracy of 96 %. Diagnostic value did not differ significantly when comparing imaging within or after 2 weeks from onset of symptoms (Table 4). In case of a prior probability of 50 % for spondylodiscitis, PPV was 94 % and NPV was 94 % for ^18^F-FDG-PET/CT. In case of a 30 % prior probability, the PPV is 90.5 % and the NPV is 98 % for PET/CT.

In a patient with a history of esophageal carcinoma, now presenting with fever and back pain, ^18^F-FDG-PET/CT showed increased metabolism at the lumbosacral region of the spine, directing to a differential diagnosis of spondylodiscitis or metastasis. CE-MRI showed a lesion of S1 and S2 with soft tissue spread into the spinal canal and around the root of S1 on the left, suspect for a metastatic neoplasm. The final clinical diagnosis was based on open-biopsy results, showing squamous cell carcinoma. ^18^F-FDG-PET/CT was scored as false positive for infection. The only two patients with false-negative results of ^18^F-FDG-PET/CT also had a false-negative CE-MRI. A second CE-MRI and a second PET/CT performed more than 2 weeks after the onset of symptoms, however, confirmed spondylodiscitis.

**Fig. 1:**
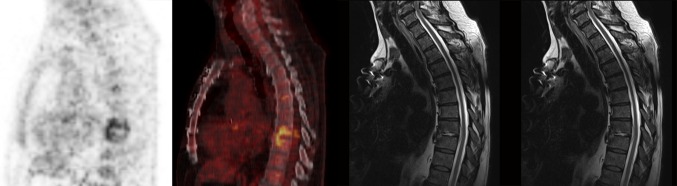
^18^F-FDG-PET/CT and MRI images of the spine in a 67-year-old man with *Streptococcus viridans* bacteraemia. ^18^F-FDG-PET/CT showed increased ^18^F-FDG-accumulation at the level Th8–Th9, correctly classified as spondylodiscitis. MRI correctly reported discitis with bulging into the spinal canal, suspect for an abscess. The patient received prolonged antibiotic treatment

Epidural or spinal abscesses were found in 15 patients (31 %) with a sensitivity for MRI of 93 and 47 % for ^18^F-FDG-PET/CT (Table 5; Fig. 1). ^18^F-FDG-PET/CT, on the other hand, showed higher sensitivity than MRI in diagnosing paravertebral abscesses (94 and 61 %, respectively), and psoas abscesses (100 and 63 %, respectively).

**Table 5:** Abscesses in patients with spondylodiscitis and sensitivity of MRI and ^18^F-FDG-PET/CT

Sensitivity for diagnosing	Total^a^	MRI^b^	^18^F-FDG-PET/CT^c^
Epidural/spinal abscess	15 (31 %)	14 (93 %)	7 (47 %)
Paravertebral abscess	18 (37 %)	11 (61 %)	17 (94 %)
Psoas abscess	8 (16 %)	5 (63 %)	8 (100 %)
Retropharyngeal abscess	3 (6 %)	2 (67 %)	3 (100 %)

Specificity was 100 % for all imaging methods, but in most cases no other diagnostic procedures were performed to confirm the found abscesses in another way

^a^Patients with abscesses at different locations are accounted for in both groups. There were 44 abscesses found in 31 patients

^b^In two patients a psoas abscess and a lumbar paravertebral abscess were missed because only cervical MRI was performed

^c^One epidural abscess initially missed in both MR imaging and ^18^F-FDG-PET/CT

### Treatment and outcome

**Fig. 2:**
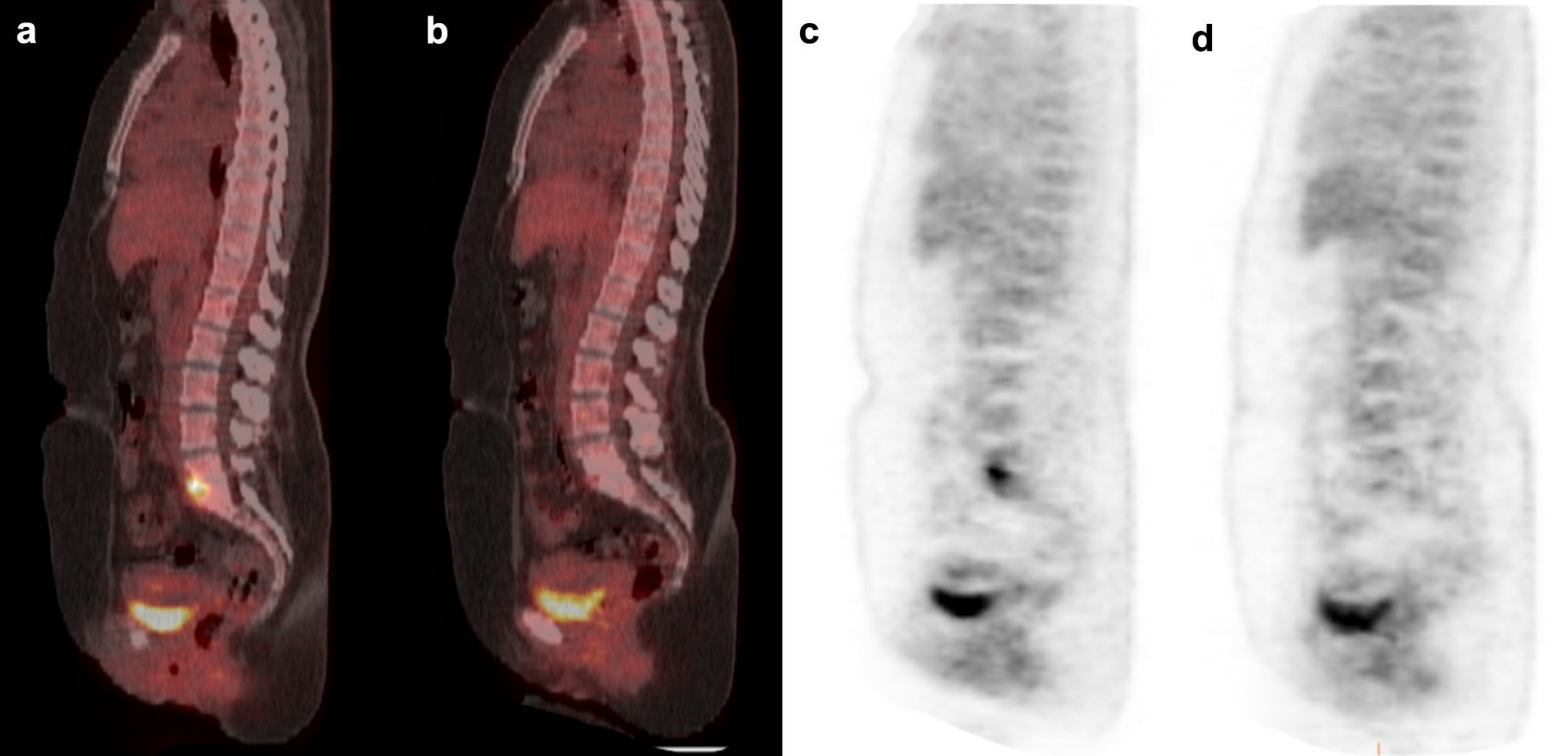
^18^F-FDG-PET/CT images before and after therapy in a 35-year-old woman with Candidemia (*Candida albicans*). ^18^F-FDG-PET/CT showed increased ^18^F-FDG-accumulation at the level L5–S1 (**a**, **c**), spondylodiscitis was confirmed by spinal biopsy which also showed *Candida albicans*. The ^18^F-FDG-PET/CT was repeated after 5 months of therapy with fluconazole and showed only minimal ^18^F-FDG-uptake (**b**, **d**) which was interpreted as reactive changes after treatment for spondylodiscitis

All patients with spondylodiscitis received antibiotic treatment. Median duration of antibiotic treatment was 124 days, with a median of 46 days of intravenous therapy followed by oral administration of median 79 days (Fig. 2). Five patients (10 %) underwent spinal surgery after the diagnosis of spondylodiscitis, because they developed progressive neurological impairment due to spinal cord compression.

The median duration of follow-up was 401 days (range 76–2340 days). At the last follow-up, six out of all 49 patients with spondylodiscitis showed neurological impairment (13 %), two patients experienced an episode of relapse (4 %), and eight patients died (17 %). Two patients died of an infection-related cause (4 % of all patients with spondylodiscitis): one during the first episode of spondylodiscitis (40 days after presentation) and one patient 4 days after admission due to relapsing spondylodiscitis with the same causative organism (*S. aureus*), 8 months after the end of the first period of treatment of spondylodiscitis. One patient with terminal heart failure died from a respiratory tract infection, 29 months after the end of adequate treatment of spondylodiscitis. Another patient died of recurrent bacteraemia with different microorganisms, due to bowel failure and chronic graft-versus-host disease, 19 months after the end of adequate treatment of spondylodiscitis. In four patients the cause of death could not be ascertained. They died 9, 10, 22, and 27 months after the end of adequate treatment of spondylodiscitis, without signs of persistent infection or relapse of spondylodiscitis.

## Discussion

In this study we retrospectively evaluated the diagnostic value of ^18^F-FDG-PET/CT and MRI in diagnosing spondylodiscitis and its complications in a cohort of 68 patients. Our study showed a significantly better overall sensitivity, NPV, and accuracy of ^18^F-FDG-PET/CT for diagnosing spondylodiscitis when compared to MRI, especially when imaging was performed within the first 2 weeks after onset of symptoms. Previous studies showing a higher diagnostic value of MRI might be based on findings of late MRI, but time from the start of symptoms until performance of MR imaging was not reported in these studies [5–7]. Modic et al. described 37 patients with suspected spondylodiscitis, of which 23 received a final diagnosis of spondylodiscitis [5]. In 14 patients this diagnosis was confirmed through biopsy results, but in nine patients the diagnosis was solely based on blood cultures or clinical evaluation. In this study, however, MRI was performed without the use of contrast agents. Early abnormalities, such as bone marrow edema are usually atypical [11]. Particularly in this type of infections, gadolinium enhancement could improve accuracy of MRI, due to increased vascularization [12, 13]. Nevertheless, the use of MRI as modality of choice is mainly based on the results of the study of Modic et al. [5]. In our study, early MRI was false negative in 11 patients despite gadolinium contrast in 9 patients. Despite the fact that CE-MRI was not performed in all patients, diagnostic outcomes of MRI showed no significant difference when comparing patients with and without contrast enhancement. Dunbar et al. described four cases of clinically suspected spondylodiscitis with early CE-MR imaging showing atypical abnormalities that were attributed to degenerative changes [14], as was the case in our study. Repeated CE-MRI 8 to 22 days later confirmed spondylodiscitis in all four cases. In line with our data, Carragee et al. showed in a group of 103 patients with spondylodiscitis that sensitivity of MRI was 55 % when imaging was performed within 2 weeks after onset of symptoms compared to 76 % after 2 weeks of symptoms [15]. In this study it was not reported whether contrast was used or not.

Three prospective studies (using stand-alone PET, not integrated PET/CT) have suggested superiority, or at least equivalent results, of ^18^F-FDG-PET over MRI in diagnosing spondylodiscitis, but could not find significant differences between these imaging modalities, as they included only small numbers of patients [6, 9, 10]. Schmitz et al. showed a sensitivity of ^18^F-FDG-PET of 100 % in 16 patients suspected of spondylodiscitis in whom the final diagnosis was based on histopathology (*n* = 12) [9]. MRI was performed in 15 patients and showed equivalent diagnostic value when compared to ^18^F-FDG-PET. In 16 patients suspected of spondylodiscitis, Gratz et al. showed a sensitivity of 100 % and a specificity of 87 % in diagnosing spondylodiscitis with ^18^F-FDG-PET, with high reliability in both low and high grade infection [6]. In this study, MRI showed a sensitivity of 82 % and a specificity of 85 %, with most false-negative results in patients with low-grade spondylodiscitis [6]. In one patient, ^18^F-FDG-PET showed an extensive paravertebral abscess, which was not seen on MRI. Stumpe et al. evaluated the use of ^18^F-FDG-PET in differentiating spondylodiscitis and degenerative abnormalities in 30 patients as compared to MRI [10]. Only four patients had a final diagnosis of spondylodiscitis. ^18^F-FDG-PET showed a sensitivity and specificity of 100 % in diagnosing spondylodiscitis compared to a sensitivity of 50 % and a specificity of 96 % in MRI. In only 14 out of 30 patients, a contrast-enhanced MR was performed. However, the results of their study implied that ^18^F-FDG-PET would be useful in differentiating between spondylodiscitis and degenerative disease. A more recent study by Fuster et al. described the value of integrated ^18^F-FDG-PET/CT compared to bone scan and ^67^Ga (but not MRI) in the diagnosis of spondylodiscitis [16]. An accuracy of 88 % was found for ^18^F-FDG-PET/CT, with a sensitivity of 89 % and specificity of 88 %. Another recent study of Fuster et al. investigated the diagnostic value of ^18^F-FDG-PET/CT and MRI in 26 patients with spondylodiscitis [17]. Sensitivity, specificity, PPV, NPV, and accuracy were 83, 88, 94, 70, and 84 % for ^18^F-FDG-PET/CT, and 94, 38, 77, 75, and 81 % for MRI, respectively. A limitation of this study was the low number of patients. In contrast to our study, these studies did not evaluate the effect of early and late imaging on the diagnostic value of ^18^F-FDG-PET/CT and MRI. In fact, none of the studies reported the time interval between start of symptoms and image acquisition.

Recently, a meta-analysis of diagnostic data on the value of FDG-PET in spondylodiscitis was performed [18]. The authors reported a sensitivity of 97 % and specificity of 88 %. However, this study analyzed mainly FDG-PET studies (*n* = 9) rather than FDG-PET/CT studies (*n* = 3).

MRI is established as the modality of choice in diagnosing epidural abscesses [7, 19], while there are no studies on the accuracy of ^18^F-FDG-PET/CT in epidural abscesses. Indeed, in our study, MRI detected nearly all epidural and spinal abscesses, while ^18^F-FDG-PET/CT was negative in approximately half of the cases, supporting the current diagnostic standards. In diagnosing paravertebral and psoas abscesses, however, sensitivity of ^18^F-FDG-PET/CT was higher than that of MRI. Solid conclusions are difficult to draw, since in three patients the abscesses were located outside the scan range. Besides several case reports, little information is available on the diagnostic value of ^18^F-FDG-PET/CT and MRI in paravertebral and psoas abscesses [6, 7, 20].

Specificity remains an issue when using ^18^F-FDG-PET/CT, in particular because it can be very difficult to reliably distinguish infection from malignancy. In our study, one ^18^F-FDG-PET/CT scored as false positive in a patient with a spinal metastasis. Nevertheless, nine of 50 patients with spondylodiscitis had a history of malignancy and ^18^F-FDG-PET/CT correctly diagnosed spondylodiscitis in eight of these patients with high specificity (95 %) in this subgroup. ^18^F-FDG-PET/CT may become a promising alternative in patients in whom MRI is contraindicated due to the presence of metallic implants in the spine or certain pacemakers. Even when spinal implants are MRI-compatible, they can give rise to artifact and limit diagnostic capability. De Winter et al. showed the value of ^18^F-FDG-PET in excluding spondylodiscitis in patients with metallic implants and after spinal surgery (sensitivity 100 %) [21]. Specificity was 65 % in patients with a metallic implant. In ^18^F-FDG-PET/CT, whole body imaging provides the opportunity to identify septic foci elsewhere, possibly even in identifying endocarditis (which appears relatively often simultaneously with spondylodiscitis) [22].

There are some limitations to our study. First, inherent to the retrospective nature of this study, it might be subjected to bias. In order to reduce bias, however, patients diagnosed with spondylodiscitis on only MRI or ^18^F-FDG-PET/CT, and patients suspected of this diagnosis with more than a 14-day delay between both imaging modalities were not included. Furthermore, images were interpreted in the clinical setting by different physicians; no revision by a single reader had been performed in order to reduce bias and to simulate the clinical setting, since revision could affect interpretation based on progressive insight during hospitalization. This could be an explanation for the fact that diagnostic values of subsequent MRI imaging improved. This study was multi-center, which entails the use of different scanners, acquisition and reconstruction parameters. In our study, 13 of 68 MRI images were performed without intravenous injection of gadolinium. Nowadays, it is recommended to perform contrast-enhanced MRI in patients suspected of spondylodiscitis.

In all participating centers, ^18^F-FDG-PET/CT has become the diagnostic standard to evaluate (high risk) patients with a Gram-positive bacteraemia systematically regarding metastatic infections [8]. This explains the high number of patients with Gram-positive bacteraemia included in the study, which may also be the reason for the high percentage of patients in whom a causative organism was identified (94 %) as compared to other studies (40–60 %) [1, 3, 23].

In patients with Gram-positive bacteraemia, cost-effectiveness of routine ^18^F-FDG-PET/CT has already been shown [8]. Whether this also holds true for other patients suspected of spondylodiscitis should be the subject of future studies. In the Netherlands, mean costs for MRI of the spine and ^18^F-FDG-PET/CT are 325 euro and 900 euro, respectively. Evaluation of the response to antibiotic treatment was outside the scope of our study, but is of high interest. Treatment effect is usually evaluated based on clinical condition and CRP levels. MRI abnormalities may persist despite clinical improvement and therefore cannot be used in early treatment evaluation [24]. Preliminary results have shown that ^18^F-FDG-PET/CT may be a promising modality for evaluation of treatment effect, which warrants further study [25]. Glaudemans et al. already addressed the possible advantages of the usage of simultaneous PET/MRI in suspected spondylodiscitis and in follow-up during treatment [26]. In our opinion this might be an excellent combination of the diagnostic value of ^18^F-FDG-PET for spondylodiscitis and the epidural and spinal abscess detection of MRI.

## Conclusion

^18^F-FDG-PET/CT shows superior diagnostic value in the early course of spondylodiscitis when compared to MRI. After 2 weeks of symptoms both modalities have similar yield. MRI showed highest sensitivity in diagnosing epidural and spinal abscesses while ^18^F-FDG-PET/CT was more sensitive in diagnosing paravertebral and psoas abscesses.
